# Hexakis(1*H*-imidazole-κ*N* ^3^)nickel(II) bis­(3-thienylacetate)

**DOI:** 10.1107/S1600536808006363

**Published:** 2008-03-14

**Authors:** Wen-Dong Song, Li-Li Ji, Hao Wang

**Affiliations:** a College of Science, Guang Dong Ocean University, Zhanjiang 524088, People’s Republic of China

## Abstract

In the title complex, [Ni(C_3_H_4_N_2_)_6_](C_6_H_5_O_2_S)_2_, the Ni^II^ atom displays an octa­hedral coordination geometry, defined by six N atoms from the imidazole ligands. Inter­molecular N—H⋯O hydrogen-bonding inter­actions between the cationic complex and 3-thienylacetate anions form a three-dimensional network architecture. The two 3-thienylacetate anions are disordered, with occupancy ratios of *circa* 0.774 (1):0.226 (1) and *ca* 0.753 (5):0.247 (5).

## Related literature

For related literature, see: Ng *et al.* (2001 ▶).
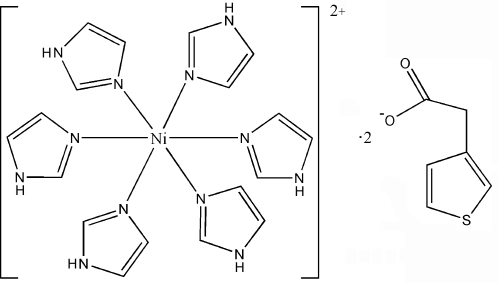


## Experimental

### 

#### Crystal data

[Ni(C_3_H_4_N_2_)_6_](C_6_H_5_O_2_S)_2_
*M* *_r_* = 749.52
Triclinic, 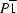

*a* = 9.2483 (3) Å
*b* = 9.8529 (3) Å
*c* = 19.6365 (6) Å
α = 84.696 (1)°
β = 88.380 (2)°
γ = 80.157 (2)°
*V* = 1755.30 (9) Å^3^
*Z* = 2
Mo *K*α radiation
μ = 0.72 mm^−1^
*T* = 296 (2) K
0.20 × 0.16 × 0.11 mm

#### Data collection

Bruker APEXII area-detector diffractometer
Absorption correction: multi-scan (*SADABS*; Sheldrick, 2004 ▶) *T*_min_ = 0.869, *T*_max_ = 0.924
13333 measured reflections
7140 independent reflections
5337 reflections with *I* > 2σ(*I*)
*R*_int_ = 0.025

#### Refinement

*R*[*F* ^2^ > 2σ(*F* ^2^)] = 0.040
*wR*(*F* ^2^) = 0.111
*S* = 1.06
7140 reflections
480 parameters
38 restraints
H-atom parameters constrained
Δρ_max_ = 0.39 e Å^−3^
Δρ_min_ = −0.27 e Å^−3^

### 

Data collection: *APEX2* (Bruker, 2004 ▶); cell refinement: *SAINT* (Bruker, 2004 ▶); data reduction: *SAINT*; program(s) used to solve structure: *SHELXS97* (Sheldrick, 2008 ▶); program(s) used to refine structure: *SHELXL97* (Sheldrick, 2008 ▶); molecular graphics: *SHELXTL* (Sheldrick, 2008 ▶); software used to prepare material for publication: *SHELXTL*.

## Supplementary Material

Crystal structure: contains datablocks I, global. DOI: 10.1107/S1600536808006363/sg2218sup1.cif

Structure factors: contains datablocks I. DOI: 10.1107/S1600536808006363/sg2218Isup2.hkl

Additional supplementary materials:  crystallographic information; 3D view; checkCIF report

## Figures and Tables

**Table 1:** Hydrogen-bond geometry (Å, °)

*D*—H⋯*A*	*D*—H	H⋯*A*	*D*⋯*A*	*D*—H⋯*A*
N12—H12⋯O4^i^	0.86	2.56	3.124 (3)	124
N12—H12⋯O3^i^	0.86	1.97	2.826 (3)	170
N10—H10*A*⋯O3^ii^	0.86	1.88	2.718 (3)	164
N8—H8*A*⋯O2^iii^	0.86	1.81	2.660 (3)	170
N4—H4*A*⋯O4^iv^	0.86	1.89	2.688 (3)	153
N2—H2⋯O1^v^	0.86	1.91	2.749 (3)	166
N6—H6⋯O1^ii^	0.86	1.90	2.711 (3)	156

Symmetry codes: (i) 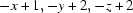; (ii) 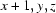; (iii) 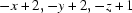; (iv) 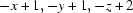; (v) 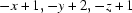.

**Fig. 1.**
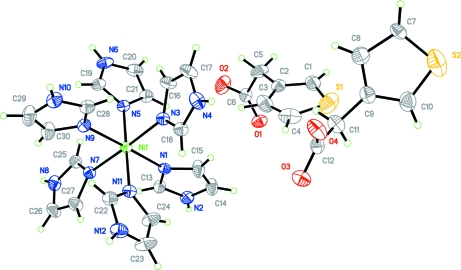
The structure of (I), showing the atomic numbering scheme. Non-H atoms are shown with 30% probability displacement ellipsoids.

**Fig. 2.**
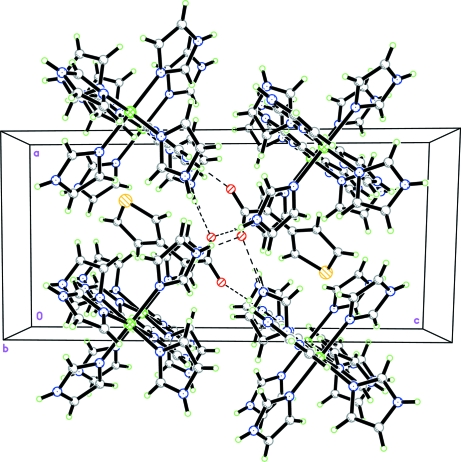
A packing view of the title compound. The intermolecluar hydrogen bonds are shown as dashed lines.

## supplementary crystallographic information

### Comment

In the structural investigation of 3-thienylacetate complexes, it has been found that the 3-thienylacetate functions as a multidentate ligand [Ng *et al.* (2001)], with versatile binding and coordination modes. In this study, we expected to obtain a complex composed of nickel(II), 3-thienylacetate and imidazole by hydrothermal reaction. Unfortunately, the Ni^II^ atom was not coordinated by 3-thienylacetate. We finally obtained the title structure, (I), composed of cations and anions.

As shown in Fig. 1, the crystal structure of the title complex consists of [Ni(C_3_H_4_N_2_)_6_]^2+^ and two different 3-thienylacetate anions. The Ni^II^ atom is coordinated by six different imidazole molecules in a slightly distorted octahedral geometry. The cationic complexes link the 3-thienylacetate anions by intermolecular N—H···O hydrogen bonding interactions (table 1) to form a three-dimensional network structure (Fig. 2).

### Experimental

A mixture of nickel chloride (1 mmol), 3-thienylacetic acid (1 mmol), imidazole (1 mmol), NaOH (1.5 mmol) and H_2_O (12 ml) was placed in a 23 ml Teflon reactor, which was heated to 433 K for three days and then cooled to room temperature at a rate of 10 K h^-1^. The crystals obtained were washed with water and dryed in air.

### Refinement

Two independent 3-thienylacetate anions are disordered and they are split into two sets of positions, with occupancy ratios of 0.774 (1):0.226 (1) and 0.753 (5):0.247 (5), respectively. Due to the significant overlap of the disordered atoms the following restraints were applied: The two rings C1 C2 C3 C4 S1 (and ring C7 C8 C9 C10 S2) and their disordered counterparts were each restrained to be flat and their equivalent bond distances were restrained to be the same within a standard deviation of 0.01 Å. All H atoms were placed at calculated positions and were treated as riding on the parent C atoms with C—H = 0.93 Å (aromatic ring), and 0.97 Å (methylene); N—H = 0.86 Å, and with *U*_iso_(H) = 1.2 *U*_eq_(C, N).

### Crystal data

**Table:**

[Ni(C_3_H_4_N_2_)_6_](C_6_H_5_O_2_S)_2_	*Z* = 2
*M**_r_* = 749.52	*F*_000_ = 780
Triclinic, *P*1	*D*_x_ = 1.418 Mg m^−^^3^
Hall symbol: -P 1	Mo *K*α radiation λ = 0.71073 Å
*a* = 9.2483 (3) Å	Cell parameters from 5680 reflections
*b* = 9.8529 (3) Å	θ = 1.4–28.0º
*c* = 19.6365 (6) Å	µ = 0.73 mm^−^^1^
α = 84.6960 (10)º	*T* = 296 (2) K
β = 88.380 (2)º	Block, blue
γ = 80.157 (2)º	0.20 × 0.16 × 0.11 mm
*V* = 1755.30 (9) Å^3^	

### Data collection

**Table:**

Bruker APEXII area-detector diffractometer	7140 independent reflections
Radiation source: fine-focus sealed tube	5337 reflections with *I* > 2σ(*I*)
Monochromator: graphite	*R*_int_ = 0.025
*T* = 296(2) K	θ_max_ = 26.5º
φ and ω scans	θ_min_ = 1.0º
Absorption correction: multi-scan(SADABS; Sheldrick, 2004)	*h* = −11→11
*T*_min_ = 0.869, *T*_max_ = 0.925	*k* = −5→12
13333 measured reflections	*l* = −24→24

### Refinement

**Table:**

Refinement on *F*^2^	Secondary atom site location: difference Fourier map
Least-squares matrix: full	Hydrogen site location: inferred from neighbouring sites
*R*[*F*^2^ > 2σ(*F*^2^)] = 0.040	H-atom parameters constrained
*wR*(*F*^2^) = 0.111	*w* = 1/[σ^2^(*F*_o_^2^) + (0.0423*P*)^2^ + 0.8694*P*] where *P* = (*F*_o_^2^ + 2*F*_c_^2^)/3
*S* = 1.06	(Δ/σ)_max_ = 0.001
7140 reflections	Δρ_max_ = 0.39 e Å^−^^3^
480 parameters	Δρ_min_ = −0.27 e Å^−^^3^
38 restraints	Extinction correction: none
Primary atom site location: structure-invariant direct methods	

### Special details

**Table:**

Geometry. All e.s.d.'s (except the e.s.d. in the dihedral angle between two l.s. planes) are estimated using the full covariance matrix. The cell e.s.d.'s are taken into account individually in the estimation of e.s.d.'s in distances, angles and torsion angles; correlations between e.s.d.'s in cell parameters are only used when they are defined by crystal symmetry. An approximate (isotropic) treatment of cell e.s.d.'s is used for estimating e.s.d.'s involving l.s. planes.
Refinement. Refinement of *F*^2^ against ALL reflections. The weighted *R*-factor *wR* and goodness of fit *S* are based on *F*^2^, conventional *R*-factors *R* are based on *F*, with *F* set to zero for negative *F*^2^. The threshold expression of *F*^2^ > σ(*F*^2^) is used only for calculating *R*-factors(gt) *etc*. and is not relevant to the choice of reflections for refinement. *R*-factors based on *F*^2^ are statistically about twice as large as those based on *F*, and *R*- factors based on ALL data will be even larger.

### Fractional atomic coordinates and isotropic or equivalent isotropic displacement parameters (Å^2^)

**Table:**

	*x*	*y*	*z*	*U*_iso_*/*U*_eq_	Occ. (<1)
C1	0.4131 (4)	0.4287 (3)	0.66425 (18)	0.0627 (9)	
H1	0.3888	0.3694	0.6339	0.075*	
C2	0.5286 (3)	0.4973 (3)	0.65584 (14)	0.0484 (7)	
C3	0.5372 (4)	0.5773 (4)	0.71015 (18)	0.0681 (9)	
H3	0.6112	0.6302	0.7111	0.082*	
C5	0.6323 (4)	0.4848 (4)	0.59608 (18)	0.0710 (10)	
H5A	0.6163	0.4062	0.5727	0.085*	
H5B	0.7315	0.4641	0.6135	0.085*	
C6	0.6230 (3)	0.6089 (3)	0.54369 (13)	0.0474 (7)	
C8	0.3563 (4)	0.2371 (3)	0.99004 (16)	0.0598 (8)	
H8	0.4549	0.2448	0.9920	0.072*	
C9	0.2493 (3)	0.3502 (3)	0.96818 (14)	0.0500 (7)	
C10	0.1119 (4)	0.3137 (4)	0.97049 (19)	0.0683 (9)	
H10	0.0270	0.3748	0.9576	0.082*	
C11	0.2791 (4)	0.4927 (3)	0.94645 (14)	0.0559 (8)	
H11A	0.1976	0.5421	0.9192	0.067*	
H11B	0.3661	0.4849	0.9174	0.067*	
C12	0.3015 (3)	0.5780 (3)	1.00487 (13)	0.0427 (6)	
C13	0.7099 (3)	1.1011 (3)	0.59500 (13)	0.0444 (6)	
H13	0.7758	1.1596	0.5804	0.053*	
C14	0.5169 (4)	1.0069 (4)	0.60100 (17)	0.0663 (9)	
H14	0.4254	0.9851	0.5928	0.080*	
C15	0.6119 (3)	0.9449 (3)	0.64942 (17)	0.0637 (9)	
H15	0.5958	0.8722	0.6808	0.076*	
C16	0.9193 (3)	0.6510 (3)	0.75680 (15)	0.0517 (7)	
H16	0.9882	0.6223	0.7234	0.062*	
C17	0.8696 (4)	0.5679 (3)	0.80681 (16)	0.0601 (8)	
H17	0.8984	0.4727	0.8145	0.072*	
C18	0.7630 (3)	0.7782 (3)	0.81614 (13)	0.0446 (6)	
H18	0.7026	0.8539	0.8327	0.053*	
C19	1.1837 (3)	0.8033 (3)	0.63842 (14)	0.0460 (7)	
H19	1.2480	0.8292	0.6684	0.055*	
N6	1.2259 (3)	0.7234 (3)	0.58772 (11)	0.0532 (6)	
H6	1.3145	0.6887	0.5772	0.064*	
C21	0.9893 (3)	0.7778 (3)	0.58834 (13)	0.0478 (7)	
H21	0.8912	0.7835	0.5772	0.057*	
C22	0.8396 (3)	1.1189 (3)	0.84289 (14)	0.0485 (7)	
H22	0.9326	1.0865	0.8606	0.058*	
C23	0.6170 (4)	1.2253 (4)	0.83415 (17)	0.0744 (11)	
H23	0.5270	1.2794	0.8429	0.089*	
C24	0.6547 (3)	1.1572 (3)	0.77818 (16)	0.0613 (9)	
H24	0.5936	1.1560	0.7415	0.074*	
C25	1.0829 (3)	1.1481 (3)	0.60810 (14)	0.0462 (6)	
H25	1.1209	1.0723	0.5843	0.055*	
C26	1.0367 (4)	1.3572 (3)	0.63700 (17)	0.0623 (8)	
H26	1.0349	1.4516	0.6382	0.075*	
C27	0.9656 (4)	1.2747 (3)	0.68025 (16)	0.0554 (8)	
H27	0.9057	1.3040	0.7168	0.066*	
C28	1.1230 (3)	0.8441 (3)	0.83343 (13)	0.0452 (6)	
H28	1.0629	0.7789	0.8455	0.054*	
C29	1.2965 (4)	0.9652 (4)	0.83702 (17)	0.0705 (10)	
H29	1.3772	1.0002	0.8507	0.085*	
C30	1.2145 (3)	1.0096 (4)	0.78075 (16)	0.0600 (8)	
H30	1.2302	1.0813	0.7487	0.072*	
N1	0.7348 (2)	1.0039 (2)	0.64586 (10)	0.0367 (5)	
N2	0.5794 (3)	1.1062 (2)	0.56680 (11)	0.0480 (6)	
H2	0.5425	1.1624	0.5332	0.058*	
N3	0.8519 (2)	0.7861 (2)	0.76277 (10)	0.0391 (5)	
N4	0.7707 (3)	0.6476 (3)	0.84356 (12)	0.0549 (6)	
H4A	0.7214	0.6201	0.8783	0.066*	
N5	1.0399 (2)	0.8411 (2)	0.64094 (10)	0.0390 (5)	
C20	1.1030 (4)	0.7072 (3)	0.55600 (15)	0.0567 (8)	
H20	1.0984	0.6565	0.5187	0.068*	
N7	0.9954 (2)	1.1416 (2)	0.66199 (10)	0.0389 (5)	
N8	1.1108 (3)	1.2744 (3)	0.59158 (13)	0.0551 (6)	
H8A	1.1657	1.2993	0.5583	0.066*	
N9	1.1042 (2)	0.9323 (2)	0.77832 (10)	0.0419 (5)	
N10	1.2373 (3)	0.8589 (3)	0.86970 (12)	0.0527 (6)	
H10A	1.2682	0.8107	0.9068	0.063*	
N11	0.7967 (2)	1.0899 (2)	0.78359 (10)	0.0405 (5)	
N12	0.7343 (3)	1.2000 (3)	0.87473 (12)	0.0579 (7)	
H12	0.7404	1.2305	0.9140	0.069*	
Ni1	0.91924 (3)	0.96550 (3)	0.711416 (15)	0.03369 (11)	
O1	0.5032 (2)	0.6882 (2)	0.53592 (10)	0.0561 (5)	
O2	0.7362 (3)	0.6167 (3)	0.50974 (13)	0.0884 (8)	
O3	0.2792 (2)	0.7082 (2)	0.99273 (10)	0.0554 (5)	
O4	0.3444 (3)	0.5178 (2)	1.06095 (10)	0.0628 (6)	
C4	0.4398 (14)	0.5770 (17)	0.7597 (8)	0.089 (3)	0.774 (12)
H4	0.4352	0.6252	0.7985	0.107*	0.774 (12)
S1	0.3174 (3)	0.4661 (3)	0.73853 (14)	0.0805 (17)	0.774 (12)
C7	0.3090 (15)	0.1157 (12)	1.0081 (8)	0.067 (3)	0.753 (5)
H7	0.3670	0.0318	1.0226	0.080*	0.753 (5)
S2	0.1132 (3)	0.1476 (2)	0.99890 (12)	0.0834 (8)	0.753 (5)
S1'	0.3871 (14)	0.5542 (15)	0.7623 (6)	0.124 (12)	0.226 (12)
C4'	0.324 (3)	0.429 (3)	0.7164 (11)	0.060 (8)	0.226 (12)
H4'	0.2484	0.3778	0.7267	0.072*	0.226 (12)
C7'	0.098 (3)	0.1816 (18)	0.9903 (14)	0.16 (2)	0.247 (5)
H7'	0.0132	0.1426	0.9924	0.193*	0.247 (5)
S2'	0.2812 (15)	0.0985 (12)	1.0115 (8)	0.091 (4)	0.247 (5)

### Atomic displacement parameters (Å^2^)

**Table:**

	*U*^11^	*U*^22^	*U*^33^	*U*^12^	*U*^13^	*U*^23^
C1	0.064 (2)	0.055 (2)	0.069 (2)	−0.0105 (16)	−0.0164 (18)	0.0026 (16)
C2	0.0470 (16)	0.0488 (17)	0.0432 (15)	0.0005 (13)	−0.0061 (12)	0.0146 (13)
C3	0.085 (3)	0.063 (2)	0.059 (2)	−0.0222 (18)	−0.0127 (19)	0.0047 (17)
C5	0.065 (2)	0.065 (2)	0.068 (2)	0.0153 (17)	0.0114 (17)	0.0182 (18)
C6	0.0476 (16)	0.0587 (18)	0.0361 (14)	−0.0126 (14)	0.0016 (12)	0.0004 (13)
C8	0.063 (2)	0.059 (2)	0.0593 (19)	−0.0130 (16)	0.0103 (15)	−0.0139 (16)
C9	0.0613 (19)	0.0543 (18)	0.0395 (15)	−0.0195 (15)	0.0042 (13)	−0.0138 (13)
C10	0.066 (2)	0.071 (2)	0.072 (2)	−0.0202 (19)	−0.0022 (18)	−0.0153 (19)
C11	0.077 (2)	0.0567 (19)	0.0371 (15)	−0.0195 (16)	−0.0023 (14)	−0.0053 (13)
C12	0.0457 (15)	0.0502 (17)	0.0345 (14)	−0.0154 (12)	0.0054 (11)	−0.0035 (12)
C13	0.0444 (15)	0.0462 (16)	0.0408 (15)	−0.0085 (12)	−0.0051 (12)	0.0078 (12)
C14	0.0580 (19)	0.069 (2)	0.074 (2)	−0.0256 (17)	−0.0246 (17)	0.0182 (18)
C15	0.061 (2)	0.062 (2)	0.070 (2)	−0.0281 (16)	−0.0207 (16)	0.0278 (17)
C16	0.0685 (19)	0.0358 (15)	0.0474 (16)	−0.0021 (13)	0.0075 (14)	−0.0018 (13)
C17	0.090 (2)	0.0334 (16)	0.0547 (18)	−0.0088 (15)	0.0014 (17)	0.0036 (14)
C18	0.0506 (16)	0.0429 (16)	0.0381 (14)	−0.0062 (12)	0.0021 (12)	0.0033 (12)
C19	0.0461 (16)	0.0480 (16)	0.0391 (14)	0.0020 (12)	0.0026 (12)	0.0020 (12)
N6	0.0540 (15)	0.0539 (15)	0.0418 (13)	0.0140 (12)	0.0104 (11)	0.0011 (11)
C21	0.0533 (17)	0.0492 (17)	0.0400 (15)	−0.0046 (13)	−0.0008 (13)	−0.0071 (13)
C22	0.0545 (17)	0.0491 (17)	0.0415 (15)	−0.0043 (13)	0.0022 (13)	−0.0113 (13)
C23	0.072 (2)	0.081 (3)	0.057 (2)	0.0263 (19)	0.0033 (18)	−0.0153 (18)
C24	0.0593 (19)	0.069 (2)	0.0482 (17)	0.0155 (16)	−0.0044 (14)	−0.0168 (15)
C25	0.0505 (16)	0.0413 (16)	0.0462 (16)	−0.0102 (12)	0.0024 (13)	0.0024 (12)
C26	0.075 (2)	0.0413 (18)	0.074 (2)	−0.0226 (16)	0.0059 (18)	−0.0003 (16)
C27	0.066 (2)	0.0464 (18)	0.0539 (18)	−0.0133 (15)	0.0080 (15)	−0.0028 (14)
C28	0.0494 (16)	0.0452 (16)	0.0384 (14)	−0.0024 (12)	−0.0073 (12)	0.0010 (12)
C29	0.063 (2)	0.097 (3)	0.056 (2)	−0.032 (2)	−0.0206 (16)	0.0054 (19)
C30	0.0579 (19)	0.075 (2)	0.0492 (17)	−0.0245 (17)	−0.0101 (14)	0.0100 (16)
N1	0.0421 (12)	0.0319 (11)	0.0344 (11)	−0.0026 (9)	−0.0028 (9)	0.0007 (9)
N2	0.0514 (14)	0.0482 (14)	0.0413 (12)	−0.0034 (11)	−0.0130 (11)	0.0067 (11)
N3	0.0439 (12)	0.0386 (12)	0.0331 (11)	−0.0054 (9)	−0.0008 (9)	0.0021 (9)
N4	0.0746 (17)	0.0543 (16)	0.0373 (12)	−0.0229 (13)	0.0035 (12)	0.0090 (11)
N5	0.0402 (12)	0.0418 (13)	0.0332 (11)	−0.0036 (9)	0.0021 (9)	−0.0008 (9)
C20	0.076 (2)	0.0509 (18)	0.0393 (15)	0.0030 (15)	0.0036 (15)	−0.0110 (13)
N7	0.0437 (12)	0.0353 (12)	0.0372 (11)	−0.0081 (9)	−0.0018 (9)	0.0016 (9)
N8	0.0532 (15)	0.0600 (17)	0.0530 (15)	−0.0209 (12)	0.0030 (12)	0.0104 (13)
N9	0.0428 (12)	0.0468 (13)	0.0348 (11)	−0.0055 (10)	−0.0040 (9)	−0.0005 (10)
N10	0.0574 (15)	0.0593 (16)	0.0387 (13)	−0.0028 (12)	−0.0140 (11)	0.0014 (11)
N11	0.0475 (13)	0.0378 (12)	0.0344 (11)	−0.0039 (10)	0.0026 (9)	−0.0011 (9)
N12	0.0740 (18)	0.0554 (16)	0.0435 (14)	−0.0027 (13)	0.0027 (13)	−0.0164 (12)
Ni1	0.03781 (18)	0.03297 (18)	0.02871 (17)	−0.00365 (13)	0.00005 (12)	0.00110 (12)
O1	0.0570 (13)	0.0541 (13)	0.0480 (11)	0.0056 (10)	0.0028 (9)	0.0148 (10)
O2	0.0612 (15)	0.118 (2)	0.0812 (17)	−0.0209 (14)	0.0233 (13)	0.0226 (16)
O3	0.0763 (14)	0.0448 (12)	0.0438 (11)	−0.0058 (10)	−0.0097 (10)	−0.0023 (9)
O4	0.1051 (18)	0.0539 (13)	0.0348 (11)	−0.0320 (12)	−0.0040 (11)	0.0037 (9)
C4	0.099 (5)	0.095 (5)	0.071 (5)	−0.018 (4)	−0.002 (4)	−0.001 (4)
S1	0.0613 (11)	0.093 (3)	0.077 (2)	−0.0054 (11)	0.0176 (11)	0.024 (2)
C7	0.069 (4)	0.065 (6)	0.068 (5)	−0.020 (4)	0.014 (3)	−0.008 (4)
S2	0.0988 (18)	0.0813 (11)	0.0849 (12)	−0.0531 (10)	0.0155 (10)	−0.0206 (9)
S1'	0.18 (2)	0.109 (13)	0.056 (4)	0.037 (15)	0.015 (7)	0.012 (5)
C4'	0.069 (15)	0.063 (16)	0.054 (16)	−0.033 (13)	0.005 (11)	0.008 (10)
C7'	0.08 (2)	0.25 (5)	0.12 (2)	0.03 (2)	−0.046 (17)	0.05 (2)
S2'	0.141 (11)	0.072 (4)	0.073 (4)	−0.055 (5)	0.026 (5)	−0.017 (3)

### Geometric parameters (Å, °)

**Table:**

C1—C4'	1.299 (15)	N6—H6	0.8600
C1—C2	1.357 (4)	C21—C20	1.336 (4)
C1—S1	1.721 (4)	C21—N5	1.386 (3)
C1—H1	0.9300	C21—H21	0.9300
C2—C3	1.395 (4)	C22—N11	1.312 (3)
C2—C5	1.495 (4)	C22—N12	1.330 (4)
C3—C4	1.307 (12)	C22—H22	0.9300
C3—S1'	1.735 (9)	C23—N12	1.339 (4)
C3—H3	0.9300	C23—C24	1.347 (4)
C5—C6	1.516 (4)	C23—H23	0.9300
C5—H5A	0.9700	C24—N11	1.368 (4)
C5—H5B	0.9700	C24—H24	0.9300
C6—O2	1.235 (3)	C25—N7	1.318 (3)
C6—O1	1.245 (3)	C25—N8	1.323 (4)
C8—C7	1.356 (12)	C25—H25	0.9300
C8—C9	1.401 (4)	C26—C27	1.355 (4)
C8—S2'	1.651 (10)	C26—N8	1.357 (4)
C8—H8	0.9300	C26—H26	0.9300
C9—C10	1.377 (5)	C27—N7	1.372 (4)
C9—C11	1.498 (4)	C27—H27	0.9300
C10—C7'	1.350 (15)	C28—N9	1.320 (3)
C10—S2	1.677 (4)	C28—N10	1.327 (3)
C10—H10	0.9300	C28—H28	0.9300
C11—C12	1.522 (4)	C29—C30	1.353 (4)
C11—H11A	0.9700	C29—N10	1.364 (4)
C11—H11B	0.9700	C29—H29	0.9300
C12—O4	1.243 (3)	C30—N9	1.377 (4)
C12—O3	1.265 (3)	C30—H30	0.9300
C13—N1	1.315 (3)	N1—Ni1	2.127 (2)
C13—N2	1.334 (3)	N2—H2	0.8600
C13—H13	0.9300	N3—Ni1	2.130 (2)
C14—N2	1.338 (4)	N4—H4A	0.8600
C14—C15	1.344 (4)	N5—Ni1	2.103 (2)
C14—H14	0.9300	C20—H20	0.9300
C15—N1	1.360 (4)	N7—Ni1	2.125 (2)
C15—H15	0.9300	N8—H8A	0.8600
C16—C17	1.342 (4)	N9—Ni1	2.149 (2)
C16—N3	1.384 (3)	N10—H10A	0.8600
C16—H16	0.9300	N11—Ni1	2.133 (2)
C17—N4	1.340 (4)	N12—H12	0.8600
C17—H17	0.9300	C4—S1	1.783 (13)
C18—N3	1.318 (3)	C4—H4	0.9300
C18—N4	1.339 (3)	C7—S2	1.796 (12)
C18—H18	0.9300	C7—H7	0.9300
C19—N5	1.319 (3)	S1'—C4'	1.782 (14)
C19—N6	1.335 (3)	C4'—H4'	0.9300
C19—H19	0.9300	C7'—S2'	1.792 (14)
N6—C20	1.351 (4)	C7'—H7'	0.9300
			
C4'—C1—C2	126.7 (11)	C27—C26—N8	106.2 (3)
C2—C1—S1	111.0 (3)	C27—C26—H26	126.9
C4'—C1—H1	108.8	N8—C26—H26	126.9
C2—C1—H1	124.5	C26—C27—N7	109.7 (3)
S1—C1—H1	124.5	C26—C27—H27	125.2
C1—C2—C3	111.1 (3)	N7—C27—H27	125.2
C1—C2—C5	123.2 (3)	N9—C28—N10	112.4 (3)
C3—C2—C5	125.7 (3)	N9—C28—H28	123.8
C4—C3—C2	119.1 (7)	N10—C28—H28	123.8
C2—C3—S1'	105.0 (6)	C30—C29—N10	106.4 (3)
C4—C3—H3	120.4	C30—C29—H29	126.8
C2—C3—H3	120.4	N10—C29—H29	126.8
S1'—C3—H3	134.6	C29—C30—N9	109.6 (3)
C2—C5—C6	117.1 (3)	C29—C30—H30	125.2
C2—C5—H5A	108.0	N9—C30—H30	125.2
C6—C5—H5A	108.0	C13—N1—C15	104.1 (2)
C2—C5—H5B	108.0	C13—N1—Ni1	126.26 (18)
C6—C5—H5B	108.0	C15—N1—Ni1	129.40 (18)
H5A—C5—H5B	107.3	C13—N2—C14	106.7 (2)
O2—C6—O1	126.1 (3)	C13—N2—H2	126.7
O2—C6—C5	115.1 (3)	C14—N2—H2	126.7
O1—C6—C5	118.7 (3)	C18—N3—C16	104.6 (2)
C7—C8—C9	116.7 (6)	C18—N3—Ni1	128.34 (18)
C9—C8—S2'	110.8 (6)	C16—N3—Ni1	125.55 (18)
C7—C8—H8	121.6	C18—N4—C17	107.4 (2)
C9—C8—H8	121.6	C18—N4—H4A	126.3
S2'—C8—H8	127.5	C17—N4—H4A	126.3
C10—C9—C8	110.9 (3)	C19—N5—C21	104.2 (2)
C10—C9—C11	124.2 (3)	C19—N5—Ni1	126.72 (18)
C8—C9—C11	124.9 (3)	C21—N5—Ni1	129.02 (18)
C7'—C10—C9	119.1 (12)	C21—C20—N6	107.1 (3)
C9—C10—S2	113.1 (3)	C21—C20—H20	126.5
C7'—C10—H10	117.4	N6—C20—H20	126.5
C9—C10—H10	123.4	C25—N7—C27	104.4 (2)
S2—C10—H10	123.4	C25—N7—Ni1	128.32 (19)
C9—C11—C12	114.9 (2)	C27—N7—Ni1	127.25 (19)
C9—C11—H11A	108.6	C25—N8—C26	107.2 (2)
C12—C11—H11A	108.6	C25—N8—H8A	126.4
C9—C11—H11B	108.6	C26—N8—H8A	126.4
C12—C11—H11B	108.6	C28—N9—C30	104.6 (2)
H11A—C11—H11B	107.5	C28—N9—Ni1	125.81 (19)
O4—C12—O3	123.2 (3)	C30—N9—Ni1	128.84 (19)
O4—C12—C11	119.3 (3)	C28—N10—C29	107.0 (2)
O3—C12—C11	117.4 (2)	C28—N10—H10A	126.5
N1—C13—N2	112.3 (2)	C29—N10—H10A	126.5
N1—C13—H13	123.9	C22—N11—C24	104.6 (2)
N2—C13—H13	123.9	C22—N11—Ni1	128.08 (19)
N2—C14—C15	106.8 (3)	C24—N11—Ni1	127.36 (18)
N2—C14—H14	126.6	C22—N12—C23	107.2 (3)
C15—C14—H14	126.6	C22—N12—H12	126.4
C14—C15—N1	110.2 (3)	C23—N12—H12	126.4
C14—C15—H15	124.9	N5—Ni1—N7	89.81 (8)
N1—C15—H15	124.9	N5—Ni1—N1	90.40 (8)
C17—C16—N3	109.3 (3)	N7—Ni1—N1	89.69 (8)
C17—C16—H16	125.4	N5—Ni1—N3	89.52 (8)
N3—C16—H16	125.4	N7—Ni1—N3	177.57 (8)
N4—C17—C16	107.3 (3)	N1—Ni1—N3	92.65 (8)
N4—C17—H17	126.4	N5—Ni1—N11	179.43 (8)
C16—C17—H17	126.4	N7—Ni1—N11	90.73 (8)
N3—C18—N4	111.5 (3)	N1—Ni1—N11	89.78 (8)
N3—C18—H18	124.3	N3—Ni1—N11	89.93 (8)
N4—C18—H18	124.3	N5—Ni1—N9	90.79 (8)
N5—C19—N6	111.9 (3)	N7—Ni1—N9	89.38 (8)
N5—C19—H19	124.0	N1—Ni1—N9	178.48 (8)
N6—C19—H19	124.0	N3—Ni1—N9	88.30 (8)
C19—N6—C20	107.1 (2)	N11—Ni1—N9	89.04 (8)
C19—N6—H6	126.5	C3—C4—S1	106.7 (10)
C20—N6—H6	126.5	C3—C4—H4	126.7
C20—C21—N5	109.6 (3)	S1—C4—H4	126.7
C20—C21—H21	125.2	C1—S1—C4	92.1 (5)
N5—C21—H21	125.2	C8—C7—S2	107.0 (8)
N11—C22—N12	112.0 (3)	C8—C7—H7	126.5
N11—C22—H22	124.0	S2—C7—H7	126.5
N12—C22—H22	124.0	C10—S2—C7	92.2 (4)
N12—C23—C24	106.7 (3)	C3—S1'—C4'	97.7 (11)
N12—C23—H23	126.6	C1—C4'—S1'	99.2 (15)
C24—C23—H23	126.6	C1—C4'—H4'	130.4
C23—C24—N11	109.5 (3)	S1'—C4'—H4'	130.4
C23—C24—H24	125.2	C10—C7'—S2'	104.2 (16)
N11—C24—H24	125.2	C10—C7'—H7'	127.9
N7—C25—N8	112.5 (3)	S2'—C7'—H7'	127.9
N7—C25—H25	123.7	C8—S2'—C7'	94.9 (11)
N8—C25—H25	123.7		

### Hydrogen-bond geometry (Å, °)

**Table:**

*D*—H···*A*	*D*—H	H···*A*	*D*···*A*	*D*—H···*A*
N12—H12···O4^i^	0.86	2.56	3.124 (3)	124
N12—H12···O3^i^	0.86	1.97	2.826 (3)	170
N10—H10A···O3^ii^	0.86	1.88	2.718 (3)	164
N8—H8A···O2^iii^	0.86	1.81	2.660 (3)	170
N4—H4A···O4^iv^	0.86	1.89	2.688 (3)	153
N2—H2···O1^v^	0.86	1.91	2.749 (3)	166
N6—H6···O1^ii^	0.86	1.90	2.711 (3)	156

Symmetry codes: (i) −*x*+1, −*y*+2, −*z*+2; (ii) *x*+1, *y*, *z*; (iii) −*x*+2, −*y*+2, −*z*+1; (iv) −*x*+1, −*y*+1, −*z*+2; (v) −*x*+1, −*y*+2, −*z*+1.
